# Have the Findings from Clinical Risk Prediction and Trials Any Key Messages for Safety Pharmacology?

**DOI:** 10.3389/fphys.2017.00890

**Published:** 2017-11-06

**Authors:** Jem D. Lane, Andrew Tinker

**Affiliations:** ^1^William Harvey Heart Centre, Barts and The London School of Medicine and Dentistry, London, United Kingdom; ^2^Department of Cardiac Electrophysiology, Barts Heart Centre, St Bartholomew's Hospital, London, United Kingdom

**Keywords:** arrhythmias, cardiac, torsades de pointes, anti-arrhythmia agents, cardiac ion channels, long QT

## Abstract

Anti-arrhythmic drugs are a mainstay in the management of symptoms related to arrhythmias, and are adjuncts in prevention and treatment of life-threatening ventricular arrhythmias. However, they also have the potential for pro-arrhythmia and thus the prediction of arrhythmia predisposition and drug response are critical issues. Clinical trials are the latter stages in the safety testing and efficacy process prior to market release, and as such serve as a critical safeguard. In this review, we look at some of the lessons to be learned from approaches to arrhythmia prediction in patients, clinical trials of drugs used in the treatment of arrhythmias, and the implications for the design of pre-clinical safety pharmacology testing.

## Introduction

Cardiac arrhythmias range from the benign to the life-threatening. The former typically arise in patients with structurally and functionally normal hearts, while the latter more commonly arise in those with acquired or genetically-determined abnormalities in cardiac structure or cellular electrophysiology. The two modalities currently available to directly target arrhythmias with the aim of prevention and/or eradication are anti-arrhythmic drugs and catheter ablation. Pharmacotherapy has been around for over 100 years, with quinine one of the first to be used (Sneader, 2005), and ironically, one of the first to be associated with inducing arrhythmia (Schwartz et al., 2016). For the majority of agents in use today, efficacy was based on clinical observation rather than *a priori* understanding of molecular mechanisms. Anti-arrhythmic drugs have retained a key role in the therapy of heart rhythm disorders, despite the advent of ablation. However, their potential to cause harm through pro-arrhythmic effects has placed constraints on the use of many existing drugs, and restricted the release of new agents to the market. The “catch-22” facing such drugs is the requirement to alter cardiac electrophysiology enough, and under the right circumstances, so as to prevent or terminate arrhythmias. Yet at the same time, they must not do so too much or they risk triggering drug-induced arrhythmias. Thus, it would seem a fine balance has to be achieved. In fact, what is required is detailed knowledge of the mechanisms of the arrhythmia requiring treatment at the cellular, tissue and organ levels, and its vulnerable parameter(s) (Task Force of the Working Group on Arrhythmias of the European Society of Cardiology, 1991; Rosen and Janse, 2010). Even more problematic is that non-cardiac drugs sometimes developed for relatively benign conditions can lead to malignant ventricular arrhythmias (Bednar et al., 2002).

Estimates of the incidence of drug-induced arrhythmia require the patient to come to medical attention, and that the diagnosis be considered. The fact that many have concomitant structural heart disease makes disentangling drug-induced from endogenous arrhythmia difficult. Partly because of this, attention is focused on torsade de pointes (TdP), which outside the setting of long QT syndrome (LQTS) is rare. TdP is also characteristic of non-cardiac drug-induced long QT in patients with normal hearts. With these considerations in mind, estimates have been made (Sarganas et al., 2014).

Safety pharmacology seeks to exclude drugs with a significant risk of pro-arrhythmia. The challenge is to set the threshold at the correct level, so as to allow safe drugs to continue through development and on to the market, and this is dependent on the methods employed in risk assessment. These methods are in the process of being modified, in light of advances in our understanding of cellular electrophysiology, and the models available. In this review, we focus on anti-arrhythmic drugs, and the role of clinical data in informing our approaches to assessment of their risks of pro-arrhythmia. We adhere to the Vaughan-Williams classification system in referring to drugs by class, acknowledging its limitations.

## Key concepts in cardiac safety pharmacology

### Ion channels and cellular electrophysiology

**Figure 1:**
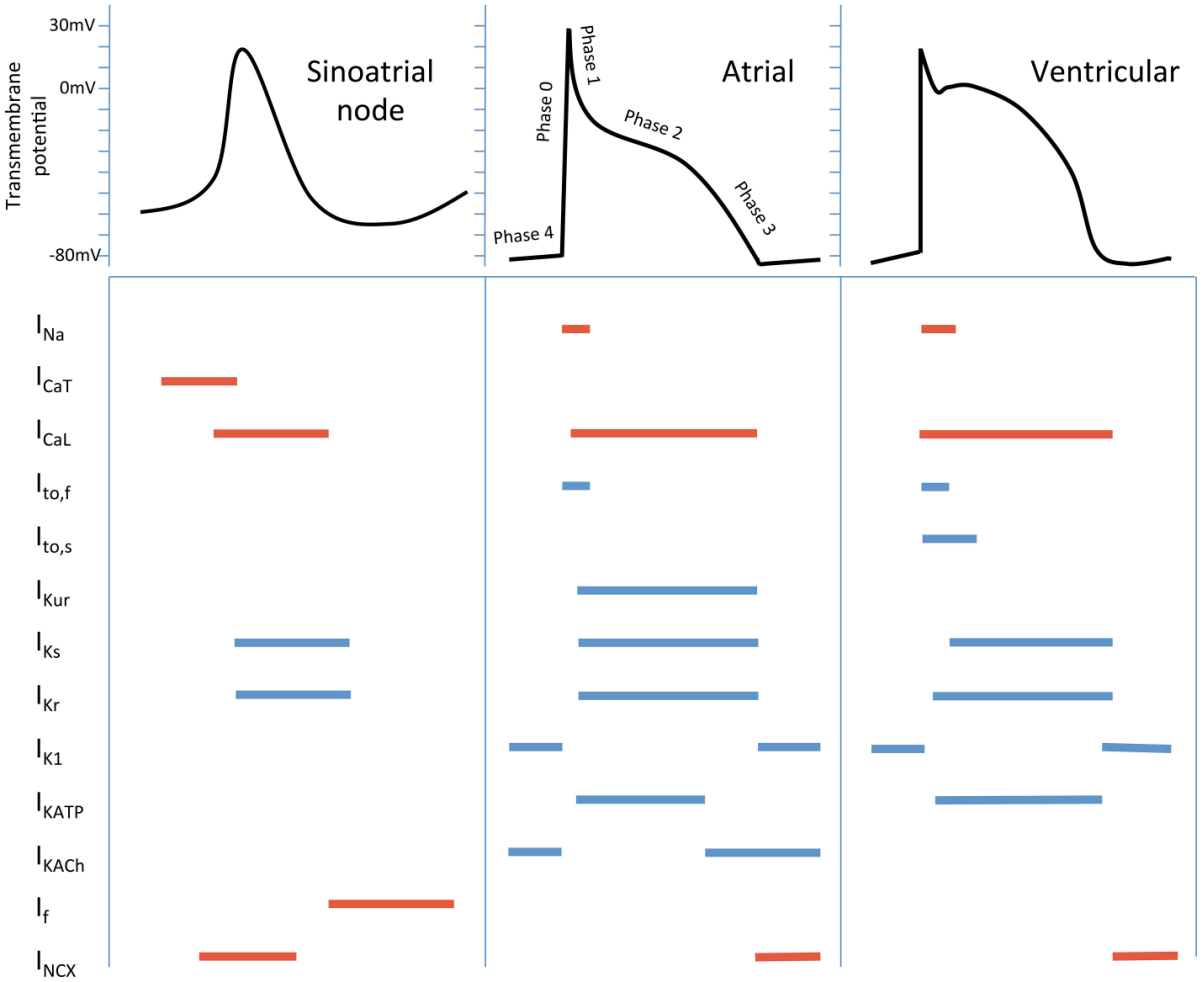
Schematic cardiac action potentials from different regions of the heart, with the currents that generate them. Colored lines indicate the phase of the action potential that the current participates in. Inward currents are in red, outward currents in blue. Currents - I_Na_, inward Na^+^; I_CaT_, T-type Ca^2+^; I_CaL_, L- type Ca^2+^; I_to,f_, fast transient outward; I_to,s_, slow transient outward; I_Kur_, ultra-rapid K^+^ delayed rectifier; I_Ks_, slow K^+^ delayed rectifier; I_Kr_, rapid K^+^ delayed rectifier; I_K1_, inward rectifier; I_KATP_, ADP-activated K^+^ channel; I_KACh_, muscarinic-gated K^+^ channel; I_f_, “funny” current; I_NCX_, Na^+^/Ca^2+^ exchange current.

Cardiomyocyte electrophysiology serves as the basis for understanding arrhythmia mechanisms, pharmacological anti-arrhythmic and pro-arrhythmic effects. The currents responsible for generating these action potentials differ in atrial and ventricular cardiomyocytes, and sinoatrial/atrioventricular nodal tissue, as well as within different regions of each chamber (Nerbonne and Kass, 2005; Grant, 2009; Figure 1).

Activation results in depolarization of the cellular membrane, which if of sufficient magnitude to attain threshold voltage, leads to generation of an action potential. This may then excite a neighboring cell via gap junctions. If the source current from one cell or group of cells is sufficient to depolarize the neighboring cells (the “sink”), propagation occurs. This cyclical process of transmembrane and intercellular ionic fluxes requires reversal of the activation process, and this is termed repolarization. Refractoriness is a distinct though closely linked concept to repolarization, and describes the state of a cell or tissue which is unexcitable, and unable to undergo depolarization.

### Mechanisms of ventricular arrhythmias

**Figure 2:**
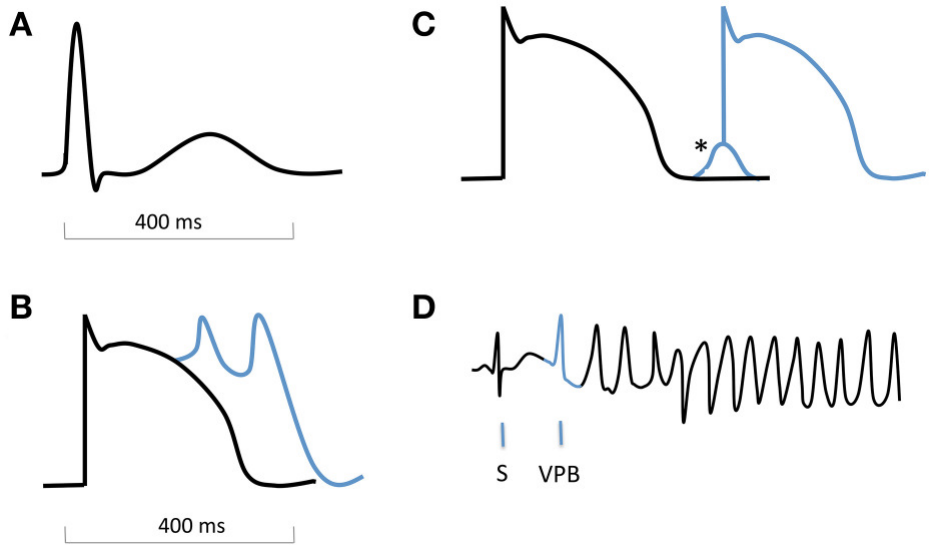
Schematic ECG, action potentials with afterdepolarizations, and onset of torsade de pointes. **(A)** normal QRS complex and T wave on an ECG. **(B)** action potential with early afterdepolarizations (EADs). **(C)** action potential with delayed afterdepolarization (DAD, ^*^). **(D)** ECG showing onset of TdP, with a sinus beat (S) followed by a ventricular premature beat (VPB, blue) which is triggered by an EAD.

Traditionally, at a cellular and tissue level these have been divided into disorders of impulse formation, disorders of conduction/propagation, or a combination of both (Zipes et al., 2005). With regards to tachyarrhythmias, the three most common mechanisms are abnormal automaticity, triggered activity and re-entry. The latter two are considered most relevant to ventricular arrhythmias. Triggered activity takes the form of either early (EADs) or delayed afterdepolarizations (DADs). EADs usually occur with delayed repolarization, which can cause “repolarization instability,” rendering cells more susceptible to premature depolarization (Shah et al., 2005). The postulated mechanisms relate either to arrest of repolarization due to diminished outward K^+^ currents, or abnormal Ca^2+^ influx, either through L-type calcium channels or the Na^+^/Ca^2+^ exchange pump (Pogwizd and Bers, 2004; Shah et al., 2005). They are best described as triggers for TdP in the setting of long QT syndrome (LQTS). DADs occur during phase 4 following completion of repolarization. They result from release of Ca^2+^ from the sarcoplasmic reticulum, which raises intracellular Ca^2+^ concentration ([Ca^2+^]i). The Na^+^/Ca^2+^ exchanger extrudes this, with resultant import of Na^+^ and a net inward current which causes premature depolarization (Nattel and Carlsson, 2006; Figure 2).

**Figure 3:**
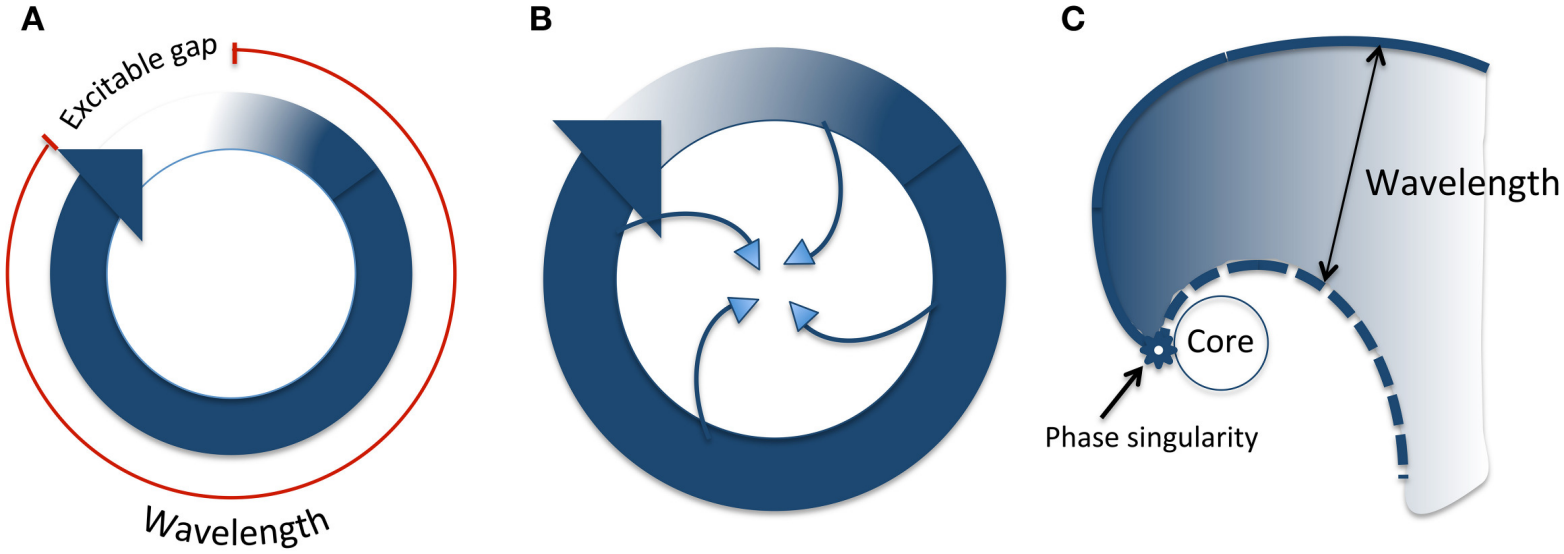
Types of re-entry. **(A)** Classical anatomical re-entry. The wavelength is the product of conduction velocity and refractory period (shown in red). The excitable gap is the section of the circuit which is unexcited, ahead of the wavefront. **(B)** Leading circle re-entry. The wavefront impinges on the wavetail such that there is no excitable gap. In addition, centripetal invasion creates a central region of functional refractoriness. **(C)** Rotor re-entry. The wavefront and wavetail meet at a phase singularity, which rotates around an unexcited core. The wavelength (distance between the wavefront and tail) varies according to distance from the phase singularity. Modified from Pandit and Jalife (2013).

Re-entry refers to a circus movement of wavefront propagation, and wavelength is defined as the product of conduction velocity and effective refractory period (ERP), and as such, it represents the length (or volume) of tissue that is refractory to new impulses. For re-entry to occur, wavelength must be shorter than the re-entrant circuit path length. The difference between these is known as the “excitable gap”—the zone of non-refractory tissue between the wavefront and wavetail. In theory therefore, prolonging wavelength beyond path length should be antiarrhythmic. Indeed, this is the mechanism of “Class III” anti-arrhythmics, though ironically, the discipline of safety pharmacology in relation to anti-arrhythmic drugs has arisen largely as a result of this effect. More complex iterations of re-entry have been proposed, incorporating functional refractoriness. In particular, the “leading circle model”, and rotors are considered important in our attempts to understand complex arrhythmias such as torsade de pointes and ventricular fibrillation (Figure 3). “Substrate” is the term used to refer to abnormal myocardium that either produces triggered activity, or by virtue of fibrosis and/or altered cellular electrophysiology, aids the creation of a path suitable for re-entry, or fosters wave break and rotor formation.

### QTc

The QT interval on the electrocardiogram (ECG) reflects the time between depolarization and repolarization of the ventricles. This interval varies with heart rate, so that a correction must be made (QTc). Measurement of the QT interval and adjustment for heart rate (utilizing the R-R interval—the time between successive QRS complexes) are deemed two of the major challenges of electrocardiography (Rautaharju et al., 2009). Various formulae are available, and while based on measurements from only 39 subjects, Bazett's correction is most commonly used (QTc = QT/√(R-R). The QT interval is relied upon as an easily accessed biomarker, reflecting repolarization. Its relevance is borne out by its prolongation in the LQTS, and its presaging TdP. Nevertheless, it has a number of shortcomings (Rautaharju et al., 2009; Sager et al., 2014).

### Repolarization reserve and risk modifiers

**Figure 4:**
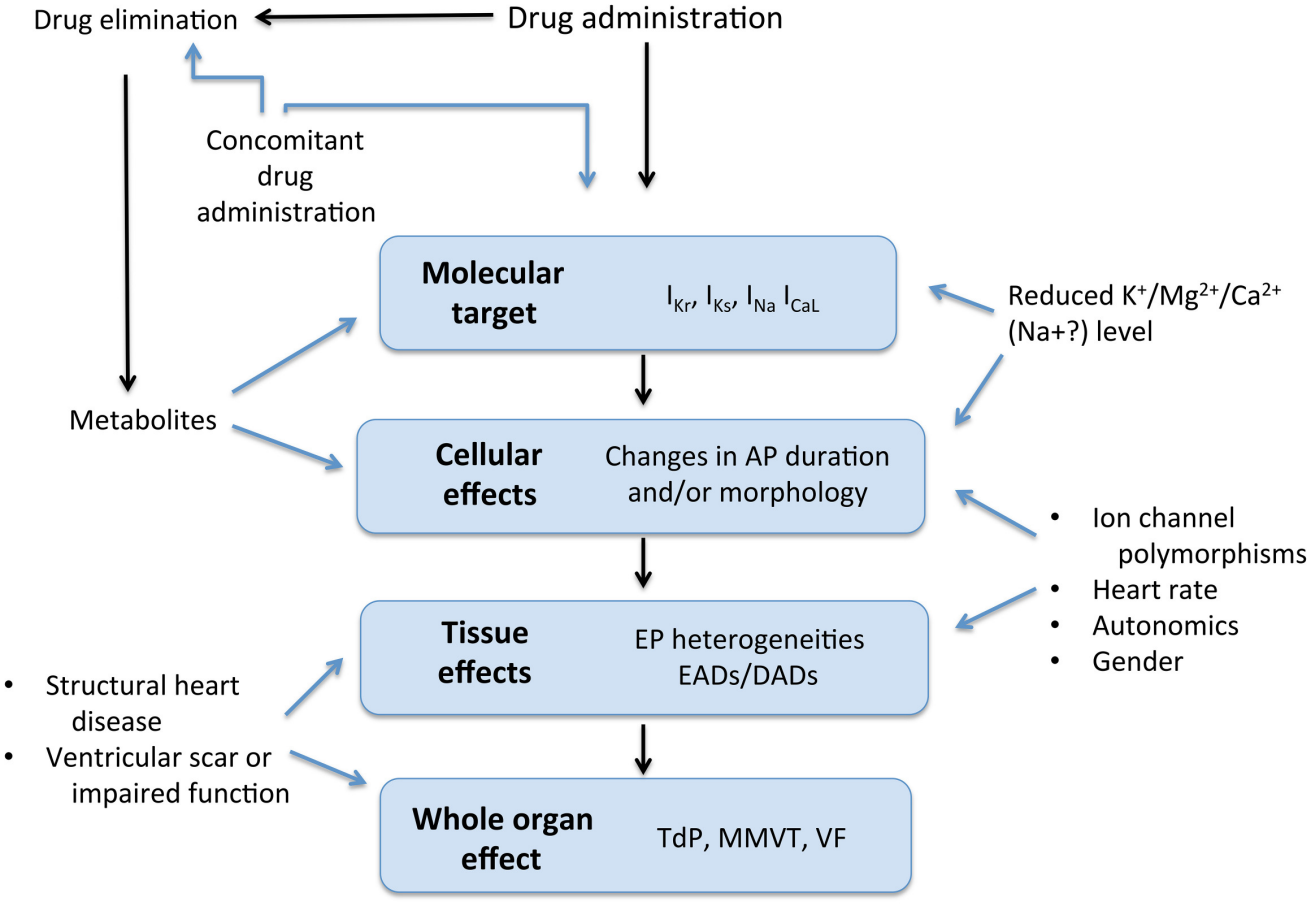
Drug effects and interactions. AP, Action potential; EP, electrophysiological; EAD, early afterdepolarization; DAD, delayed afterdepolarization; TdP, torsade de pointes; MMVT, monomorphic ventricular tachycardia; VF, ventricular fibrillation. Modified from Roden (2004).

As with most rare but serious occurrences, a single factor is rarely sufficient on its own to lead to a ventricular arrhythmia. In the case of TdP in particular, this is due to repolarization reserve. This describes a degree of redundancy among repolarizing currents, such that if one is reduced, others may compensate to a degree, maintaining action potential duration, and preventing EADs (Roden and Abraham, 2011). Nevertheless, reserve only protects up to a point, and when several factors act in concert, protection may be lost and arrhythmia may ensue. Ion channel polymorphisms with subclinical effects, impaired clearance of an ion channel-blocking drug, concurrent use of more than one drug, female sex (Makkar et al., 1993; Gaborit et al., 2010) and hormonal derangement (Lane et al., 2012) are just a few of the factors that may modify risk (Figure 4).

## Drug-induced arrhythmias

Whilst the focus of safety pharmacology for anti-arrhythmics is the potential to induce ventricular tachyarrhythmias, it is worth considering other arrhythmias that may result. For example, a number of drugs have been shown to trigger atrial fibrillation (AF) (Strickberger et al., 1997; van der Hooft et al., 2004; Kaakeh et al., 2012). For example, there are good data for adenosine, dobutamine, theophyllines and acute alcohol excess precipitating AF (Strickberger et al., 1997; van der Hooft et al., 2004; Kaakeh et al., 2012). In the setting of reduced clearance or concomitant administration, atrioventricular (AV) nodal-blocking drugs such as beta-blockers and calcium channel antagonists may induce heart block.

Ventricular arrhythmias may occur as a result of therapy with Class I agents (Falk, 1989; The Cardiac Arrhythmia Suppression Trial (CAST) Investigators, 1989; Tisdale and Miller, 2010), though this is exceedingly rarely seen in practice, likely as a result of the restriction of use of these drugs to patients without evidence of QRS or QT prolongation on the ECG, and structurally normal hearts. Closer attention has been paid to drugs that prolong the QT interval due to the risk of precipitating TdP. Probably this largely stems from the fact there are more drugs that affect repolarizing K^+^ currents than I_Na_, so the incidence of arrhythmias is higher due to more widespread use. It may be that in addition, repolarizing currents have less reserve than does I_Na_, and phase 3 of the action potential is as a result, more vulnerable.

At present, clinical practice relies largely on drug indication, ECG markers, indices of cardiac contractility, electrolyte levels and concurrent use of other medication with QT prolonging effects (Drew et al., 2010) to guide risk assessment. More accurate evidence-based scoring systems have been developed (discussed below). Of the ECG biomarkers available, QRS duration, QT interval, T wave morphology and non-sustained or sustained VT are the most easily assessed and clinically useful (Wellens et al., 2014). For example, there has been interesting work done looking at periodic oscillations in repolarization as measured using the T-wave. The authors found a low frequency oscillation <0.1 Hz associated with sympathetic activity but not heart rate variability or respiratory ventilation. It correlated strongly with outcomes after myocardial infarction (Rizas et al., 2014, 2017). However, it is complex to measure. In the absence of a more readily available and accessible measure of cardiac repolarization, the QT interval has retained its role as an important biomarker despite its many shortcomings (Hondeghem, 2006). However, even without the difficulties in measurement, reliance on this oversimplifies the assessment of drug-induced repolarization disturbance (Hondeghem, 2006).

## Pre-clinical approaches to screening

A major issue for industry is identifying cardiac risk for non-cardiac drugs: there seems to be little interest in developing new antiarrhythmic agents for ventricular arrhythmia for the reasons discussed below. However, it is also important not to exaggerate potential toxicity and discard potentially useful agents. Until recently, screening for pro-arrhythmia was based on the International Conference on Harmonization non-clinical and clinical evaluation guidelines, S7B and E14, respectively (International Conference on Harmonsation of Technical Requirements for Registration of Pharmaceuticals for Human Use, 2005a,b). Essentially, these focused on measurement of the human ether-a-go-go related gene (hERG) channel current I_Kr_, and the ECG parameter QTc, as means of identifying drugs with the potential to cause TdP. Heterologous expression systems and animal models have been central to pre-clinical screening, with guinea pig, rabbit, dog and monkey being the most utilized species (Friedrichs et al., 2005; Champeroux et al., 2015). And non-rodent models have demonstrated good correlation of *in vivo* QT measurements with those in humans (Vargas et al., 2015).

Whilst effective at excluding torsadogenic compounds from market release, proposals for a new screening paradigm have come about due to concerns about oversensitivity and low specificity for detecting pro-arrhythmic potential with the S7B/E14 guidelines, as well as a drive to reduce the number of animals involved in experiments (Lu et al., 2008; Sager et al., 2014; NC3Rs). In addition, improvements in understanding of ion channel physiology, species differences in both cardiac electrophysiology and pharmacokinetics (Haushalter et al., 2008), developments in computer modeling, and the advent of stem cell technology, have reached a stage where it is advantageous to try to incorporate them in the process. A new paradigm known as the Comprehensive *in vitro* Proarrhythmia Assay (CiPA) has therefore been proposed, and is supported by a number of national and international government and commercial bodies (CiPA project, 2000). CiPA recommends a move toward human-based approaches, with screening of multiple ion channels and computer modeling central to this. There is also the aspiration to use human induced pluripotent stem cell (iPSC) models. Overall there is a move away from the emphasis on I_Kr_ and the QT interval, due to recognition of the co-dependence and interplay of ionic currents, multichannel effects of drugs (Li et al., 2017), and the shortcomings of the QT interval and importance of other ECG parameters such as the PR and QRS intervals (Sager et al., 2014).

CiPA is still in the process of being validated (Cavero et al., 2016; Colatsky et al., 2016), and has not yet been accepted to supersede the S7B/E14 guidelines. The hope that iPSC derived cardiomyocytes can assume a confirmatory role within the framework, is ambitious and perhaps the least certain of CiPA's four components, given their relative novelty. There is a growing acceptance that these cells are immature compared to native adult myocytes and are more fetal in terms of their electrophysiology and other properties (Veerman et al., 2015; Rodriguez et al., 2016). The latter may be circumvented by use of human cardiac tissue, for example from organ donors (Page et al., 2016), though this is not without its own difficulties, chiefly the lack of availability in many countries. It may be worthwhile to calibrate findings in iPSC cardiomyocytes with those from human myocytes to validate measurements. Nevertheless, a reappraisal of existing guidelines' strengths and weaknesses, and attempts to enhance the accuracy of cardiac safety testing by making use of new techniques and improved understanding, is commendable. And importantly, the new paradigm is being systematically validated prior to implementation.

## Clinical trials on antiarrhythmic drugs

**Figure 5:**
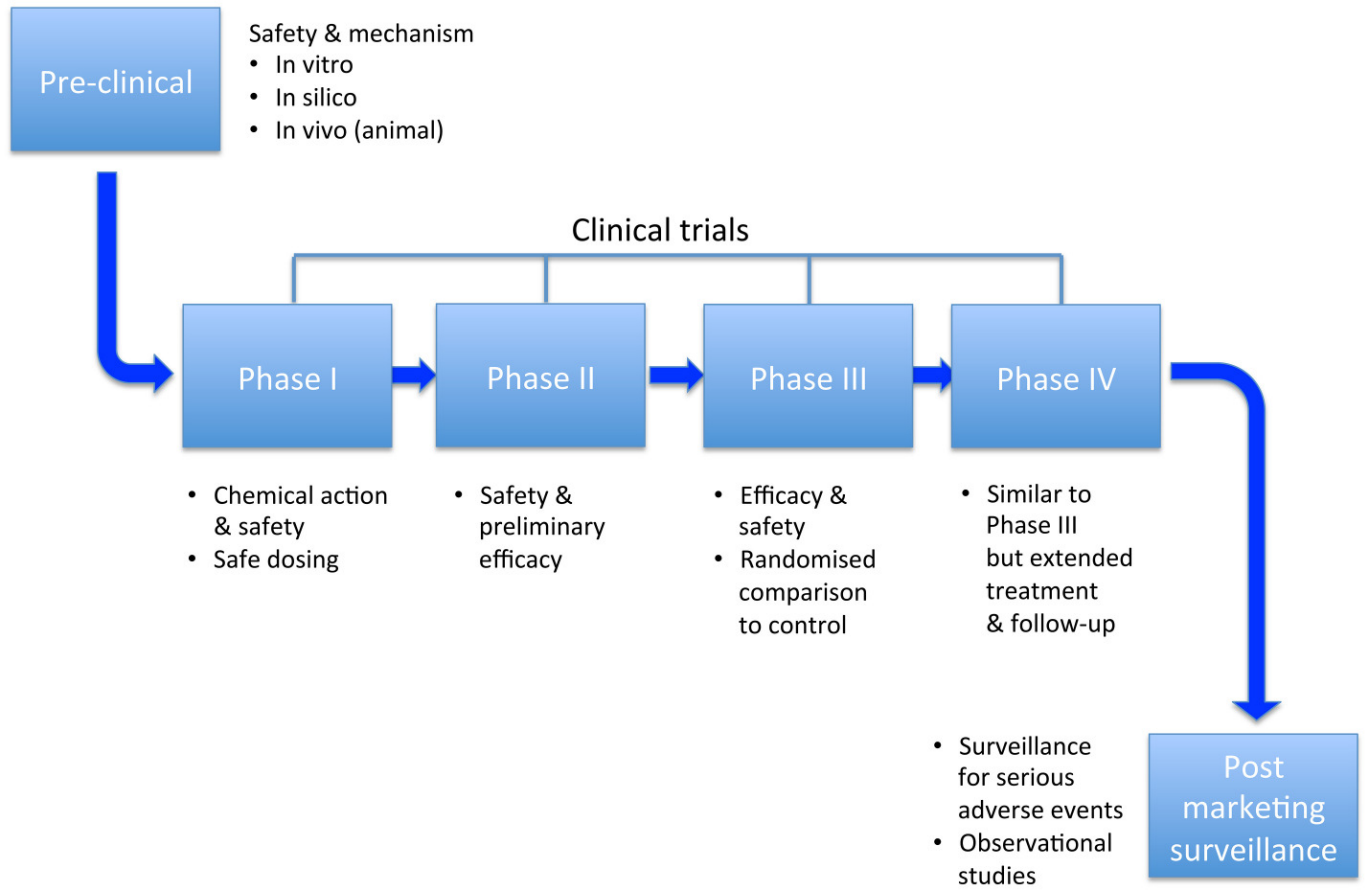
Milestones of research and development.

Clinical trials have been of paramount importance in the field of safety pharmacology for anti-arrhythmics. Unexpected findings have brought about the widespread use of beta-blockers in heart failure, and the restricted use of many other drugs such as flecainide and sotalol. They enable assessment of hard endpoints rather than surrogates, and provide opportunities to test repolarization and activation reserve *in vivo*. The main stages in this development process are illustrated in Figure 5. An overview of two of the most important trials is provided, prior to looking at the evidence relating to a number of anti-arrhythmic drugs. Rather than provide an exhaustive account of clinical trials involving anti-arrhythmic drugs, we try to focus on randomized trials that have been instructive in terms of safety, or changed practice.

### Cardiac arrhythmia suppression trial (CAST)

This landmark randomized controlled trial (RCT) was both disappointing and hugely influential. To investigate whether suppression of ventricular premature beats (VPBs) in patients following myocardial infarction (MI) reduced their risk of sudden death, patients were assigned to the Na^+^ channel/I_Na_ blockers, encainide, flecainide, moricizine, or placebo (flecainide also has some hERG/IKr-blocking effects, but I_Na_ blockade is pharmacodynamically more important). A preliminary report of the drug titration phase in 1989 revealed that despite their apparent suppression of VPBs, there was an excess of arrhythmic deaths in patients assigned to encainide or flecainide (The Cardiac Arrhythmia Suppression Trial (CAST) Investigators, 1989). This was confirmed in the full report of 1498 patients assigned to these two drugs or placebo. An excess of both arrhythmic and non-arrhythmic cardiac deaths were seen (Echt et al., 1991). A few points are noteworthy regarding the study. Firstly, beta-blocker use was low by contemporary standards: between 20 and 30% across all groups. Calcium channel blocker use, primarily diltiazem, was high (47–53%), as was digitalis (16–24%). Secondly, mean baseline left ventricular ejection fraction (LVEF) was 39–40%. The second part of the study comparing moricizine to placebo is less widely discussed, but found similar results (The Cardiac Arrhythmia Suppression Trial II Investigators, 1992). The drug was withdrawn in 2007 (Structural Bioinformatics Group at Charité, 2000). The fallout has resulted in avoidance of flecainide (and other “Class IC” drugs) in patients with “structural heart disease”—particularly ischaemic heart disease with a history of MI, but extrapolated to essentially anyone with any abnormality in ventricular structure and function. The rationale for this has been questioned (Kramer and Josephson, 2010). In terms of possible mechanisms for the observed pro-arrhythmia, slowing of conduction velocity with resultant facilitation of re-entry has been posited (Ruskin, 1989), though late development of ischaemia and accumulation of high drug levels may also have contributed (Aliot et al., 2011).

### Survival with oral d-sotalol (SWORD) trial

This RCT recruited a similar patient demographic to CAST: those with LVEF <40% and a history of MI (Waldo et al., 1996). The objective was to evaluate whether a phase 3 K^+^ channel (hERG/IKr) blocker, d-sotalol, reduced all-cause mortality compared to placebo. The trial was stopped prematurely due to an increased risk of death in the d-sotalol group (5.0 vs. 3.1%, relative risk 1.65, *p* = 0.006) (Waldo et al., 1996). This was presumed to be primarily due to arrhythmias; unfortunately beyond the fact that the risk of death was higher in women, justification for this assumption could be challenged. In terms of possible mechanisms for arrhythmic death, beta-blocker use was again low pre-randomization (32–33%), and digoxin use was high (48–50%). Importantly though, patients were initiated on 100 mg twice daily of d-sotalol, and if tolerated with a QTc < 520 ms, the dose was increased to 200 mg twice daily. Then, if this dose was tolerated with a QTc < 560 ms, patients remained on this dose for the study's duration. Such QT prolongation is well-established as a risk for TdP (Makkar et al., 1993; Drew et al., 2010), and would be inconceivable in a modern trial.

### Amiodarone

Amiodarone interacts with multiple ion channels, resulting in reduced I_Na_, I_Kr_, I_Ks_, I_CaL_, as well as antagonizing α- and β-adrenoceptors and acetylcholine receptors (Zimetbaum, 2012; Darbar, 2014). It has been studied in a large number of randomized trials in the setting of AF or ventricular arrhythmias (Doval et al., 1994; Julian et al., 1997; Roy et al., 2000; Bardy et al., 2005; Singh et al., 2005; Connolly et al., 2006; Le Heuzey et al., 2010). Paradoxically it often prolongs the QT interval, yet has long been known to have a low incidence of TdP, possibly due to its actions on inward currents (Lazzara, 1989; Vorperian et al., 1997; Roden, 2004). There is a higher risk of bradycardic events nevertheless (Vorperian et al., 1997). More recently, a meta-analysis of over 8,000 patients in RCTs comparing amiodarone to placebo or control found amiodarone was associated with a reduction in sudden cardiac death, though not a significant reduction in overall mortality (Piccini et al., 2009). Notably, in the GESICA trial it was found to confer improved survival in the setting of heart failure (Doval et al., 1994). And in the European Myocardial Infarct Amiodarone Trial (EMIAT), it was demonstrated to reduce arrhythmic deaths by 35% in those with LVEF ≤ 40%, though had no effect on all-cause or cardiac mortality (Julian et al., 1997). The lack of benefit on overall mortality was supported by SCD-HeFT (Bardy et al., 2005). Thus, it is one of the few drugs considered safe for use in patients with a history of MI and reduced LV function.

### Beta-adrenoceptor antagonists

This class of drugs exerts effects via antagonism of β1 and/or β2-adrenoceptor signaling. β1-adrenoceptors signal via the stimulatory G protein, Gs, and the cyclic adenosine monophosphate (cAMP) and protein kinase A (PKA) cascade. PKA increases I_CaL_, I_Ks_ and possibly I_Na_ (Brodde and Michel, 1999; Grant, 2009). β2-adrenoceptor signaling is more complex: it also couples to Gs, but can also be induced to couple to Gi, the inhibitory isoform (Xiao et al., 1995). This increases I_KACh_, and may negatively regulate I_CaL_ (Nagata et al., 2000; Zuberi et al., 2010). β-adrenoceptor antagonists are the most studied anti-arrhythmics, due to their use for both supraventricular and ventricular arrhythmias, as well as heart failure and hypertension (Packer et al., 1996, 2002; MERIT-HF Study Group, 1999; The Cardiac Insufficiency Bisoprolol Study II, 1999; Pedersen et al., 2014; Katritsis et al., 2017). They have been demonstrated to reduce mortality in heart failure, including the risk of sudden cardiac death (Hjalmarson, 1997; MERIT-HF Study Group, 1999; The Cardiac Insufficiency Bisoprolol Study II, 1999; Packer et al., 2002). Risk of pro-arrhythmia is essentially limited to the small risk of AV conduction block, which in the absence of overdose, severe renal dysfunction or concomitant AV nodal-blocking drug use, occurs extremely rarely.

### Dofetilide

A “pure” IKr blocker, dofetilide was investigated in patients with severe LV impairment and heart failure as a treatment for AF in the DIAMOND-CHF study (Torp-Pedersen et al., 1999). It performed better than placebo in converting patients with AF to sinus rhythm, though the rate of conversion by 1 month was low (12% vs. 1%). Maintenance of sinus rhythm was also higher for the dofetilide group. It was shown to be associated with a reduced rate of hospitalization for worsening heart failure. However, there was a 3.3% rate of TdP in those treated with the drug. A subsequent trial (DIAMOND-MI) investigated use of the drug in patients with recent MI and LV dysfunction (Køber et al., 2000). Again, there was no effect on all-cause or cardiac mortality, nor on arrhythmic deaths. It showed some efficacy in restoring sinus rhythm in those with AF, but there was a TdP event rate of approximately 1%. Further trials, predominantly in AF and atrial flutter have confirmed its anti-arrhythmic efficacy, but also its pro-arrhythmic potential (Bianconi et al., 2000; Singh et al., 2000). Thus, dofetilide exhibits reasonable anti-arrhythmic efficacy, and does not appear to increase mortality, yet there is a significant risk of TdP such that its use requires close monitoring (Abraham et al., 2015; Schwartz et al., 2016). Therefore, whilst current guidelines indicate it can be used to treat atrial flutter acutely (Katritsis et al., 2017), alternative drug therapy and catheter ablation have rendered this largely obsolete, in Europe at least.

### Dronedarone

This multichannel blocking drug is similar to amiodarone but with reduced extra-cardiac effects (Tadros et al., 2016). Despite a promising start in trials such as EURIDIS/ADONIS and ATHENA (Singh et al., 2007; Hohnloser et al., 2009), subsequent trials in patients with permanent AF and heart failure did not support its anti-arrhythmic potency, and moreover, it was associated with worsening of heart failure and increased mortality (Køber et al., 2008; Connolly et al., 2011). Nevertheless, there does not appear to be a significant pro-arrhythmic tendency, reinforcing the notion that drugs with multichannel effects and complex actions can still be safe, in this regard at least.

## Alternative and evolving clinical approaches

The preceding discussion has shown that there is room for improvement in prediction of pro-arrhythmia. At the clinical level, strategies can broadly be divided into those focusing on the drugs, and those focusing on patient factors. Haverkamp et al addressed both in 2001 (Haverkamp et al., 2001). They identified many of the clinical risk factors still in use today, and came up with what is to our knowledge the first attempt to stratify drugs according to propensity to induce TdP. The list of drugs was limited, and the classification was not developed. Around the same time, the Georgetown University Center for Education and Research on Therapeutics (GUCERT) was awarded money to investigate the potential of drugs to induce TdP. Subsequently based in Arizona and renamed, AZCERT, a not-for-profit organization published lists of drugs known to be associated with, and causative of QT prolongation and TdP at www.qtdrugs.org. Currently the lists are available at www.crediblemeds.org. Brugadadrugs.org is a similar website set up by the University of Amsterdam Academic Medical Center, providing advice on drugs to avoid, and drugs with possible therapeutic use for patients with Brugada syndrome (University of Amsterdam Academic Medical Center, 2017).

Clinical risk factors for ventricular fibrillation (VF) were evaluated by Da Costa et al in 91 patients with pause-dependent TdP in the setting of QT prolongation. LVEF, presence of structural heart disease, and an index of QT dispersion were found to be significant predictors (Da Costa et al., 2000). And clinical scoring systems based on patient factors have been developed. For example, Tisdale et al utilized data from 900 patients to develop a scoring system to predict QTc prolongation, and then validated this in 300 additional patients (Tisdale et al., 2013). Female sex, diagnosis of MI, sepsis, LV dysfunction, administration of QT-prolonging drugs, use of loop diuretics, serum K^+^ < 3.5 mEq/L and QT interval on admission >450 ms were identified as independent risk factors. The system had reasonable sensitivity and specificity. Although useful, it utilized a biomarker rather than a patient outcome as an endpoint. Such risk scores are likely to gain importance as electronic prescribing becomes more widespread, with automated alert systems also becoming more feasible (Haugaa et al., 2013).

The ultimate aim of precision medicine is to tailor treatment to the specific patient and the genetic make-up is likely to play a major role in determining this. There have been substantial efforts to understand the genomic architecture of the heritability of the QT interval in the general population. A variety of loci have been identified from genome wide association study (GWAS) findings (Arking et al., 2014). It was shown that one of the signals in the nitric oxide synthase 1 adaptor protein predicted predisposition to drug-induced long QT syndrome (Jamshidi et al., 2012). Furthermore, Strauss and colleagues created a “genetic QT” score, and investigated its ability to predict drug-induced QTc prolongation, and TdP (Strauss et al., 2017). While it was a significant predictor of both, the predictive power was modest, leaving much of the variability unaccounted for.

## Implications for safety pharmacology

The literature on the use of antiarrhythmic drugs illustrates a number of important points.

### There is no universal biomarker predicting risk

The QTc remains an important biomarker, though it is far from the only clinical marker of a drug's pro-arrhythmic risk. Clinical trials have demonstrated that amiodarone and beta-blockers remain two of the safest agents in terms of pro-arrhythmia. Their mechanisms are different, yet they both exert anti-arrhythmic effects, and cardiac contra-indications are few. Importantly, amiodarone confounds the predictive power of I_Kr_ and QTc screening, by virtue of its APD and QT-prolonging effects, with minimal associated pro-arrhythmic risk. It highlights the oversensitivity of the S7B and E14 guidelines: had it not been in use already, one of the most effective and safe (in arrhythmia terms) drugs may have been excluded from the market. Amiodarone's cardiac safety, together with flecainide's and sotalol's pro-arrhythmogenicity, serve as the strongest reminders of the current importance of clinical trials and post-marketing surveillance in bringing to light unexpected, unpredicted and counterintuitive findings; of how predictions based on theory may not be borne out in practice. But where this is the case, there is an opportunity to learn.

### Underlying patient pathology is important

The presence of pre-existing cardiac conditions such as LV impairment and ischaemic heart disease modulate risk of pro-arrhythmia, such that use of certain drugs, deemed safe in those with structurally normal hearts, is given careful consideration in patients with a history of these conditions. They, and other risk modifiers, such as female sex, diuretic use, hypokalaemia, and concomitant use of other QT-prolonging drugs, identified in clinical reports and risk models (Drew et al., 2010), must be included in *in silico* models (Wiśniowska and Polak, 2016) if computer modeling and prediction is to fully realize its potential. Identification of drugs with significant risk of arrhythmia may enable us to gain insight into the reasons for this. For example, the list of drugs available at www.crediblemeds.org may have arisen due to similarities in the behavior of the drug molecules in their interaction with ion channels, or alternatively their pharmacokinetics. Ultimately, feedback such as this to pre-clinical models, and clinical trials' validation of these models will hopefully lead, via an iterative process, to greater confidence in the predictive powers of computer, animal and stem cell models (Carusi et al., 2012), with a greater burden of safety testing and prediction occurring in these, rather than in trials in humans. Those models that cannot predict with reasonable accuracy must be honed, or discarded. Increased pre-clinical predictive accuracy should allow more compounds to reach the clinical trial stage. This, together with post-marketing surveillance, will retain a key role in highlighting unexpected findings, due to our inability to completely account for human physiology, pathophysiology, pharmacokinetics, and pharmacodynamics, as well as inter-individual variability in these factors, in any model.

### Ion channels remodel in disease and disease specific models may be necessary

Many of the experimental and computational approaches rely on the assessment of a compound against parameters or cells derived from healthy normal individuals. However, it is clear that the expression of ion channels significantly remodels in pathological states and this may account for proarrhythmia under such conditions. For example, in atrial fibrillation the expression of L-type calcium currents in the atria is reduced and this in itself generates a substrate for further atrial fibrillation (Wijffels et al., 1995; Gaspo et al., 1997; Yue et al., 1999). It is clear that ion channel remodeling also occurs in many other cardiac pathologies although is not so well defined (Nattel et al., 2007). On this background individual genetic differences are likely to modify the response (Munroe and Tinker, 2015). Thus, we may see the development of disease specific computational models and/or engineered cellular assay systems. In this regard the development of computational approaches to explore models with large numbers of varying parameters is likely to be valuable (Britton et al., 2013, 2017).

## Author contributions

Both authors have made a substantial, direct and intellectual contribution to the work, and approved it for publication.

### Conflict of interest statement

The authors declare that the research was conducted in the absence of any commercial or financial relationships that could be construed as a potential conflict of interest.
